# Improving CoQ_10_ productivity by strengthening glucose transmembrane of *Rhodobacter sphaeroides*

**DOI:** 10.1186/s12934-021-01695-z

**Published:** 2021-10-30

**Authors:** Yuying Yang, Lu Li, Haoyu Sun, Zhen Li, Zhengliang Qi, Xinli Liu

**Affiliations:** 1 Key Laboratory of Shandong Microbial Engineering, College of Bioengineering, Qilu University of Technology (Shandong Academy of Sciences), Jinan, Shandong People’s Republic of China; 2 State Key Laboratory of Bio-Based Material and Green Papermaking (LBMP), Qilu University of Technology (Shandong Academy of Sciences), Jinan, Shandong People’s Republic of China

**Keywords:** *R. sphaeroides*, Glucose transmembrane, Glucose metabolism, CoQ_10_ productivity

## Abstract

**Background:**

Several *Rhodobacter sphaeroides* have been widely applied in commercial CoQ_10_ production, but they have poor glucose use. Strategies for enhancing glucose use have been widely exploited in *R. sphaeroides*. Nevertheless, little research has focused on the role of glucose transmembrane in the improvement of production.

**Results:**

There are two potential glucose transmembrane pathways in *R. sphaeroides* ATCC 17023: the fructose specific-phosphotransferase system (PTS^Fru^, *fruAB*) and non-PTS that relied on glucokinase (*glk*). *fruAB* mutation revealed two effects on bacterial growth: inhibition at the early cultivation phase (12–24 h) and promotion since 36 h. Glucose metabolism showed a corresponding change in characteristic vs. the growth. For Δ*fruA*Δ*fruB*, maximum biomass (*Bio*_*max*_) was increased by 44.39% and the CoQ_10_ content was 27.08% more than that of the WT. *glk* mutation caused a significant decrease in growth and glucose metabolism. Over-expressing a galactose:H^+^ symporter (*galP*) in the Δ*fruA*Δ*fruB* relieved the inhibition and enhanced the growth further. Finally, a mutant with rapid growth and high CoQ_10_ titer was constructed (Δ*fruA*Δ*fruB*/*tac*::*galP*_*OP*_) using several glucose metabolism modifications and was verified by fermentation in 1 L fermenters.

**Conclusions:**

The PTS^Fru^ mutation revealed two effects on bacterial growth: inhibition at the early cultivation phase and promotion later. Additionally, biomass yield to glucose (*Y*_*b/glc*_) and CoQ_10_ synthesis can be promoted using *fruAB* mutation, and *glk* plays a key role in glucose metabolism. Strengthening glucose transmembrane via non-PTS improves the productivity of CoQ_10_ fermentation.

**Supplementary Information:**

The online version contains supplementary material available at 10.1186/s12934-021-01695-z.

## Introduction

Glucose is a common monosaccharide that is available in abundance. As glucose is cheap and easy to use for microorganisms, it can serve as an ideal source of carbon for producing high-value products, such as CoQ_10_, through microbial fermentation [1–3]. Studies related to microbial glucose metabolism have always been a hot topic in industrial microbiology [1, 4]. As glucose metabolism pathways have been well established for many microorganisms, many novel biotechnologies, particularly metabolic engineering, synthetic biology, and systems biology, have been applied to modify intracellular metabolic pathways to enhance the glucose utilization efficiency in microorganisms [1, 4, 5]. Besides the functional enzymes for glucose metabolism, glucose utilization also requires a set of genes that encode specific transporters and regulators [5]. Glucose transmembrane is an important step because exogenous glucose cannot go into cells through free diffusion and must rely on a transporter to cross the cell membrane [6]. Recently, researchers have realized the importance of glucose transport efficiency during microbial fermentation and focus on microbial sugar transmembrane studies [6–8]. So far, the sugar transmembrane mechanisms of many industrial microbes are still unknown, limiting metabolic modification of sugar transmembrane in these microbes.

Microorganisms depend on more than one system to transport exogenous glucose; the glucose transmembrane mechanisms for *E. coli* have been widely investigated [6, 7]. *E. coli* can use two pathways for glucose transmembrane: phosphoenolpyruvate (PEP): carbohydrate phosphotransferase system (PTS) and non-PTS [6]. The non-PTS include the ATP binding cassette (ABC) system and the major facilitator superfamily (MFS) system. PTS^Glc^ is a multiprotein phosphorelay system that accompanies the import and simultaneous phosphorylation of carbohydrates. Since the discovery of the PTS^Glc^ in *E. coli*, it has existed in many other bacteria [9]. It has been confirmed that the PTS^Glc^ is primarily composed of enzymes including IICB^Glc^/IIA^Glc^(EIIs), HPr, and enzyme I (EI). EI and HPr, the two sugar-nonspecific protein constituents of the PTS, are soluble cytoplasmic proteins participating in the transport of all PTS carbohydrates [6]. EIIs are sugar-specific transporters connecting the common PEP/EI/HPr phosphoryl transfer pathway. PTS^Glc^ is considered an effective way to use glucose because only one phosphoenolpyruvate is coupled with the translocation phosphorylation of glucose when forming an ATP. In contrast, use of glucose through the ABC transporter requires extra ATP for glucose phosphorylation in the carbohydrate kinase reaction [6, 10]. Therefore, PTS^Glc^ is a preferred channel for transferring exogenous glucose into cells in industrial bacteria, such as *E. coli*, *Bacillus subtilis,* and *Corynebacterium glutamicum* [4, 11, 12]. *E. coli* activates the non-PTS system for glucose transmembrane when exogenous glucose concentration is low (< 1 mM), or PTS^Glc^ function is defective [7]. Some bacteria lacking PTS^Glc^, such as *Pseudomonas putida*, utilize the ABC system to transfer exogenous glucose into cells [9].

*R. sphaeroides* has received significant attention because of its wide biotechnological applications, such as its ability to synthesize a high content of CoQ_10_, carotenoids, and isoprenoids as a source of pharmaceutical materials [13–16]. CoQ_10_ is an oil-soluble quinone that has a decaprenyl side chain. So far, it has been widely used in functional food and cosmetics industries because of its antioxidant function. Researchers have recently found that CoQ_10_ can regulate several genes that play an important role in cholesterol metabolism, inflammatory responses, or both [14]. Moreover, it is beneficial to patients with cardiovascular diseases, hypertension, and Parkinson’s disease [17]. Compared with the methods of animal and plant extraction and chemosynthesis, the production of CoQ_10_ by microbial fermentation is low-cost, safe, and efficient [16]. Additionally, *R. sphaeroides*, a CoQ_10_ producer with high contents of CoQ_10_, has been widely used in the industrial production of CoQ_10_. For the commercial production of CoQ_10_ with microbial fermentation, glucose acts as a major carbon source. Various strategies for guiding the metabolic flux toward CoQ_10_ biosynthesis have been exploited for *R. sphaeroides* [16]. Nevertheless, presently, there is little research on improving CoQ_10_ production by modifying the glucose transmembrane. Although *R. sphaeroides* has a wide spectrum of carbon source utilization, it had a low glucose consumption rate than *E. coli* [18, 19]. Fuhrer et al. reported that the glucose uptake rate of *R. sphaeroides* was 1.8 ± 0.1 mmol/g dry cells weight (DCW)/h, which was only approximately 23.07% as that of *E. coil* [19]. Therefore, the glucose transmembrane process of *R. sphaeroides* was a bottle-neck step for glucose metabolism, which may be a new way to further promote the productivity of CoQ_10_.

Considering the importance of glucose transmembrane for glucose metabolism and poor glucose utilization efficiency of *R. sphaeroides*, the first potential pathway of glucose transmembrane of *R. sphaeroides* were analyzed in this work. Later, a deep study was conducted to show the function of these pathways on glucose metabolism. Finally, we evaluated the influence of glucose transmembrane on CoQ_10_ synthesis efficiency and optimized CoQ_10_ fermentation by *R. sphaeroides* by modifying glucose transmembrane. Moreover, *R. sphaeroides* ATCC 17023 is a paradigmatic organism among isolated *R. sphaeroides* strains with clear genetic background and mature genetic manipulation tools that we chose as a research object in this study [20].

## Material and methods

### Strains, media and growth conditions

All the strains used in this study were summarized in Table 1. *R. sphaeroides* ATCC 17023 (wild type, WT) was used as the parental strain in this work. The mutant strains were constructed in this background. *E. coli* JM109 was used as a plasmid host, and *E. coli* S17-1 was used to conjugate DNA into *R. sphaeroides*. *R. sphaeroides* ATCC 17023 and mutant strains were routinely cultivated at 32℃ in medium A (3 g/L glucose, 2 g/L NaCl, 8 g/L yeast extract, 0.256 g/L MgSO_4_·7H_2_O,1.3 g/L KH_2_PO_4_, 15 μg/L biotin, 1 mg/L thiamine hydrochloride, 1 mg/L nicotinic acid, pH 7.2) as seed (exponentially phase cells, OD_600_ ≥ 2). *R. sphaeroides* cultures were incubated at 32℃ in modified Sistrom’s minimal medium (MSMM) lacking succinate and with glucose as an alternative carbon source (6.5 g/L) [18]. Before inoculation the seeds of these strains were washed with sterilized PBS (pH 7.2), and then resuspended with the same solution to adjust the cell density equal (OD_600_ 1). Antibiotics were added into the medium A and MSMM when necessary. *E. coli* JM109 and *E. coli* S17-1 were grown at 37 °C in Luria–Bertani (LB) medium with antibiotics when necessary. The concentrations of antibiotics and chemicals used in the construction of plasmids and recombinant strains were as follows: 25 μg/mL kanamycin and 150 μg/mL K_2_TeO_3_ for *R. sphaeroides*, and 100 μg/mL kanamycin for *E. coli* strains. Lab-scale fermentation of CoQ_10_ was carried out with medium B containing 40 g/L glucose, 6.3 g/L MgSO4, 4 g/L corn steep liquor, 3 g/L sodium glutamate, 3 g/L (NH4)_2_SO_4_, 3 g/L KH_2_PO_4_, 2 g/L CaCO_3_, 1 mg/L nicotinic acid, 1 mg/L thiamine hydrochloride, and 15 μg/L biotin, supplemented with 25 μg/mL kanamycin.

**Table 1:** Strains and plasmids used in this work

Strains and plasmids	Description	Reference or source
Strains		
Wild-type	*Rhodobacter sphaeroides* ATCC 17025	Lab preservation
Δ*glk*	*glk* markerless deletion mutant	This work
Δ*fruA*Δ*fruB*	*fruAB* markerless deletion mutant	This work
Δ*fruA*Δ*fruB/*bp	Δ*fruA*Δ*fruB* harboring pBBR1MCS-2	This work
Δ*fruA*Δ*fruB/galP*_*OP*_	Δ*fruA*Δ*fruB* harboring pBBR1MCS-2:: *galP*	This work
Δ*fruA*Δ*fruB*/*tac*::*galP*_*OP*_	Δ*fruA*Δ*fruB* harboring pBBR1MCS-2:: *tac*::*galP*	This work
Δ*fruA*Δ*fruB*/*tac*::*glk*_*OP*_	Δ*fruA*Δ*fruB* harboring pBBR1MCS-2:: *tac*::*glk*	This work
* E. coli* S17-1	*recA*, harboring the genes *tra*, *proA*, *thi-*1(pRP4-2-Tc::Mu-Km::Tn7)	Lab preservation
Plasmids		
pK18*mobsacB*	suicide vector, *sacB* (sucrose sensitivity), Km^r^	Lab preservation
pBBR1MCS-2	*ori* pBBR1, *lac*Za, Km^r^, used for geng over-expression	Lab preservation
pBBR1MCS-2::*tac*	pBBR1MCS-2 harboring the *tac* promoter	Lab preservation
pK18*mobsacB*::*glk*-L-R	For *glk* deletion	This work
pK18*mobsacB*::*fruA*-L-R	For *fruA* deletion	This work
pK18*mobsacB*::*fruB*-L-R	For *fruB* deletion	This work
pBBR1MCS-2::*galP*	For *galP* expression	This work
pBBR1MCS-2::*tac*::*galP*	For *galP* expression with strong promoter *tac*	This work
pBBR1MCS-2::*tac*::*glk*	For *glk* expression with strong promoter *tac*	This work

### Plasmids construction

Plasmids used in this study were listed in Table 1, and the related primers and restriction enzymes were presented in Additional file 6: Table S1. pK18*mobsacB*::*glk*-L-R was constructed with splicing by overlap extension (SOE) PCR. Briefly, an upstream and downstream fragment of *glk* was amplified with primer pair *glk*-L-F/*glk*-L-R and *glk*-R-F/*glk*-R-R using *R. sphaeroides* ATCC 17025 genomic DNA as template, respectively. The two fragments were joined by SOE PCR to generate a 1,560-bp fragment, which was digested and ligated into the *Pst*I/*Sph*I sites of pK18*mobsacB* to obtain pK18*mobsacB*::*glk*-L-R. The details of experiment were presented in Additional file 1: Fig. S1. An upstream and downstream fragment of *fruA* was amplified with primer pair *fruA*-L-F/*fruA*-L-R and *fruA*-R-F/*fruA* -R-R. The two fragments were joined by SOE PCR to generate a 1,819-bp fragment, which was digested and ligated into the *Sal*I/*Eco*RI sites of pK18*mobsacB* to obtain pK18*mobsacB*::*furA*-L-R. The construction of pK18*mobsacB*::*fruB*-L-R obeyed to the same procedure of pK18*mobsacB*::*furA*-L-R construction. The two fragments were amplified with primer pair *fruB*-L-F/*fruB*-L-R and *fruB*-R-F/*fruA*-R-R, and then were joined by SOE PCR to generate a 1399-bp fragment. Afterwards, the fragment was ligated into the *Eco*RI/*Sph*I sites of pK18*mobsacB* to obtain pK18*mobsacB*::*furB*-L-R. The details of the two plasmids construction were shown in Additional file 2: Fig. S2. The wide host-range conjugative plasmids pBBR1MCS-2 and pBBR1MCS-2::*tac* were used to construct the over-expression vectors. For the construction of pBBR1MCS-2::*tac*::*galP*, the entire open reading frame of *galP* gene was amplified by PCR with primer pair *galP*_*op*_*-*F/*galP*_*op*_*-*R using *E. coil* K-12 substr. MG1655 genomic DNA as template. After digestion with *Xba*I/*Sac*I, it was ligated into pBBR1MCS-2::*tac* to obtain pBBR1MCS-2::*tac*::*galP*. The details of pBBR1MCS-2::*tac*::*galP* construction were presented in Additional file 3: Fig. S3. Simultaneously, pBBR1MCS-2::*galP* was obtained following the same method of pBBR1MCS-2::*tac*::*galP* construction.

### Strains construction

Δ*glk* and Δ*fruA*Δ*fruB* were constructed as in-frame markerless deletion of almost the entire open reading frames by a two-step recombination strategy, which was based on diparental conjugation as previously described [14]. In short, *E. coli* S-17 bearing the target plasmid was used as the donor strain, and *R. sphaeroides* ATCC 17023 was used as the recipient strain. The donor/recipient ratio was 1:7 for conjugation and the colonies resistant to 150 μg/mL K_2_TeO_3_ and 25 μg/mL kanamycin were picked and cultivated for plasmid extraction and sequencing verification. For the construction of Δ*glk*, the pK18*mobsacB*::*glk*-L-R was transformed into *R. sphaeroides* ATCC 17023 by diparental conjugation. Clones obtained from single homologous recombination event were selected on kanamycin/K_2_TeO_3_ supplemented medium A agar plates, and then grown for 48–72 h in medium without antibiotic, followed by serial dilution and plating onto SMM plates containing 10% (w/w) sucrose to select for a second crossover event. Finally, sucrose-resistant and kanamycin-sensitive colonies were selected as potential positive transformants and verified by PCR using *glk*-P1/*glk*-P4 primers. To obtain the mutant Δ*fruA*Δ*fruB*, Δ*fruA* was firstly constructed, and then the gene *fruB* was knocked out of the genome of Δ*fruA* further. The method was similar to that of the construction of Δ*glk*. The Δ*fruA*Δ*fruB* was verified by primer pair *fruA*-P1/*fruA*-P4 and *fruB*-P1/*fruB*-P4. For the construction of Δ*fruA*Δ*fruB/tac*::*galP*_*OP*_, pBBR1MCS-2::*tac*::*galP* was transformed into Δ*fruA*Δ*fruB* by diparental conjugation, and selected on medium A supplemented with kanamycin (25 μg/mL) and K_2_TeO_3_ (150 μg/mL). Afterwards, the resistant clones were selected and verified. Also, other gene over-expression mutants were constructed following the same method. Primers used for mutant strains verification were listed in Additional file 7: Table S2.

### RNA extraction and RT-qPCR assay

Different growth-phase cells (1 × 10^7^) cultured in MSMM medium were harvested by centrifugation at 8000×*g* for 3 min at 4 ℃. Total RNA was extracted from *R. sphaeroides* using a Total RNA Extraction Kit and purified as described by the procedures. Synthesis of cDNA was performed by the reverse transcription reaction according to the HiFiScript cDNA Synthesis Kit instructions. Cham Q Universal SYBR qPCR master mix (Vazyme, Nanjing, China; Q711-02) and the ABI First Step Quantitative Polymerase Chain Reaction System (Applied Biosystems, San Mateo, CA, USA) were used to perform quantitative polymerase chain reactions under the following reaction conditions: 95 °C for 3 min, followed by 40 cycles of 95 °C for 10 s, 56 °C for 30 s, and 72 °C for 30 s. As an internal standard control, the relative abundance of 16S rRNA was used to standardize the results. All assays were performed in triplicate. Primers used in RT-qPCR were listed in Additional file 8: Table S3. The result of RT-qPCR was calculated based on the following formula:

$${{2}^{-\Delta\Delta{\mathrm{Ct}}}=2}^{-[\left(\text{Ct of Gi}/{\rm{A}}-{\text{Ct of Gc}}/{\rm{A}}\right)-\left({\text{Ct of Gi}}/{\rm{B}}-{\text{Ct of Gc}}/{\rm{B}}\right)\}},$$

where G_i/A_ is the gene of interest in sample A, G_c/A_ is the gene of internal control in sample A, G_i/B_ is the gene of interest in sample B and G_c/B_ gene of internal control in sample B.

### CoQ_10_ fermentation in Lab-scale bioreactors

For the lab-scale fermentation of CoQ_10_, three 1-L quadruple fermentation tanks (Bench-Top Bioreactor Multifors 2 type, INFORS HT, Switzerland) were used. The operation procedure was based on the method of Zhang et al. [13]. Seeds of *R. sphaeroides* were prepared as above shaking-flask cultivation. Subsequently, 16 mL of seeds were inoculated into tank with 0.8 L sterilizing medium B supplemented with 25 μg/mL kanamycin, and then, cultured at 32 °C for 96 h. The pH was maintained at 7.0. The aeration and agitation protocol were 1 vvm and 400 rpm during the whole fermentation. Bacterial growth (OD_600_), glucose concentration and CoQ_10_ titer were detected each 12 h.

### Analytical methods

Cultured broth was fetched each 12 h for biomass determination at OD_600_ by spectrophotometer (MAPADA INSTRUMENTSUV-1800, China) and calculated using a calibration curve which indicated the relationship between OD_600_ and dry cell weight (DCW) (1OD_600_ approximately equaled to 0.40 g DCW/L). In this study, 100 mL of cultivated broth were centrifugated to obtain cell pellets, and then used to get dry cells through lyopilization for DCW measuring. Residual glucose was detected by a SBA-40 Biosensor (Biology Institute of Shandong Academy of Sciences, China). pH was measured at the starting and end of cultivation by a SevenCompact™ pH meter S220 (METTLER TOLEDO, China). The method of extraction and quantification of CoQ_10_ was according to Zhang et al. [13]. In the study, all experiments were repeated three times. The data shown in the corresponding tables and figures were the mean values of the experiments and the error bars indicated the standard deviation. Data was treated via one-way ANOVA method (P < 0.05).Statistical significance was determined using the SAS statistical analysis program, version 8.01 (SAS Institute, Cary, NC, USA).

## Results

### Potential glucose transmembrane pathways for *R. sphaeroides* ATCC 17023

**Fig. 1:**
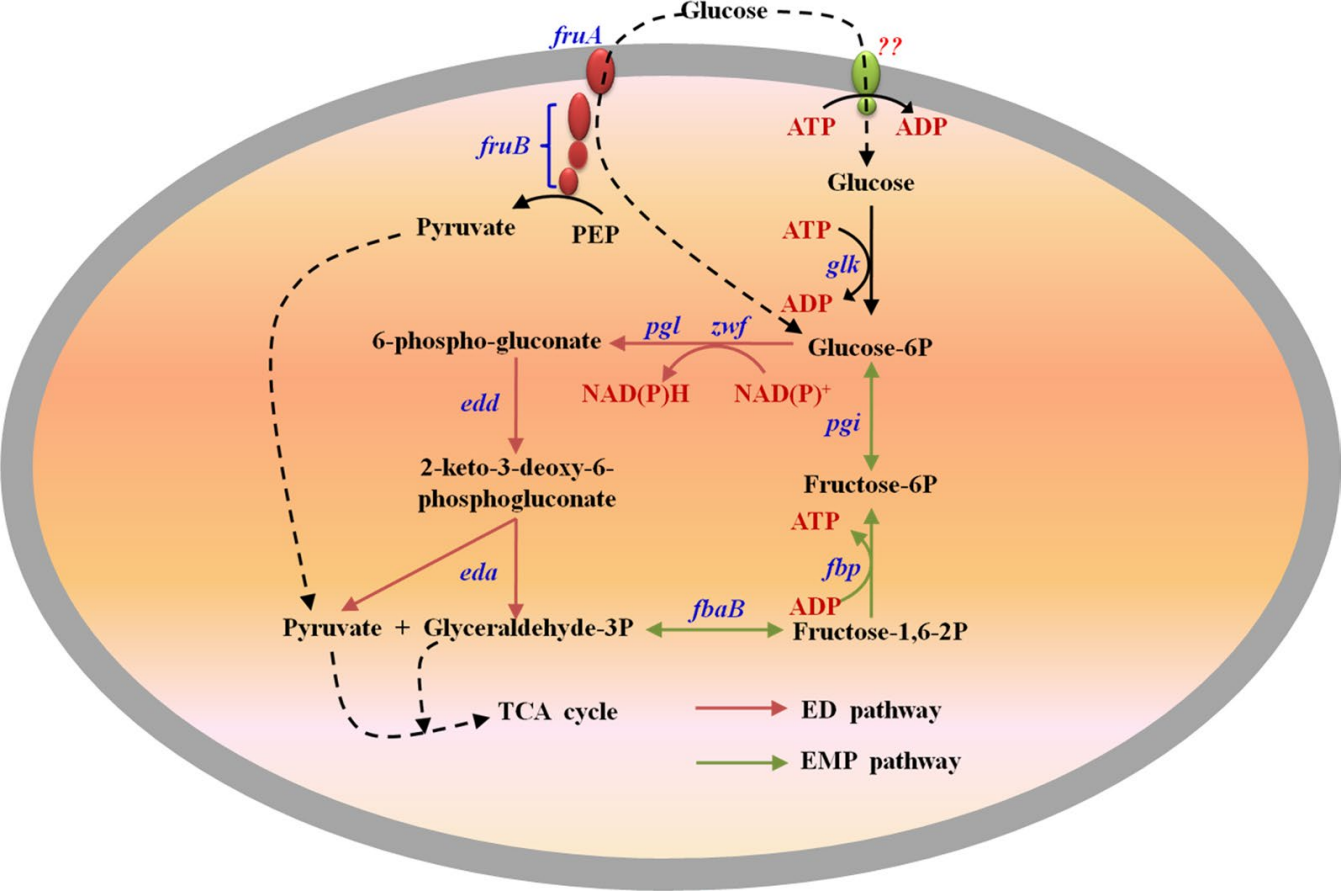
Map of potential glucose transmembrane and metabolism pathways in *R. sphaeroides* ATCC 17023

By retrieving the NCBI database, the only integral PTS, fructose-specific PTS (PTS^Fru^), was found in the genome of *R. sphaeroides* ATCC 17023, which is encoded by the gene cluster *fruAB* (RSP_1788 and RSP_1786). *fruB* encodes EI and HPr, the two sugar-nonspecific protein constituents of the PTS, and *fruA* encodes the sugar-specific transporter. It was reported that PTS^Fru^ encoded by *fruAB* simultaneously had a function of glucose transmembrane in some *E. coil* strains [6]. Additionally, *fruAB* in *R. sphaeroides* may have a similar function as that of the abovementioned *E. coil* strains. Glucose transported into cells with non-PTS must be phosphorylated before subsequent metabolism. Although the non-PTS-type glucose-specific transporter in *R. sphaeroides* ATCC 17023 has not been identified, the enzyme glucokinase (*glk*, RSP_2875), which plays a role in glucose phosphorylation, exists in the genome [5, 8]. Additionally, the glucokinase activity had been determined in some *R. sphaeroides* strains when cultured with glucose as the sole carbon source [21]. Considering the above information, *R. sphaeroides* ATCC 17023 should possess the non-PTS. *R. sphaeroides* ATCC 17023 metabolizes glucose exclusively with the Entner–Doudoroff pathway (ED) under aerobic and anaerobic conditions because of the lack of phosphofructokinase in the Embden–Meyerhof–Parnas pathway (EMP) [18]. According to the abovementioned analysis, metabolic networks that contain the glucose transmembrane and catabolism were constructed and the result is depicted in Fig. 1.

### Influence of PTS and non-PTS on bacterial growth and glucose metabolism

**Fig. 2:**
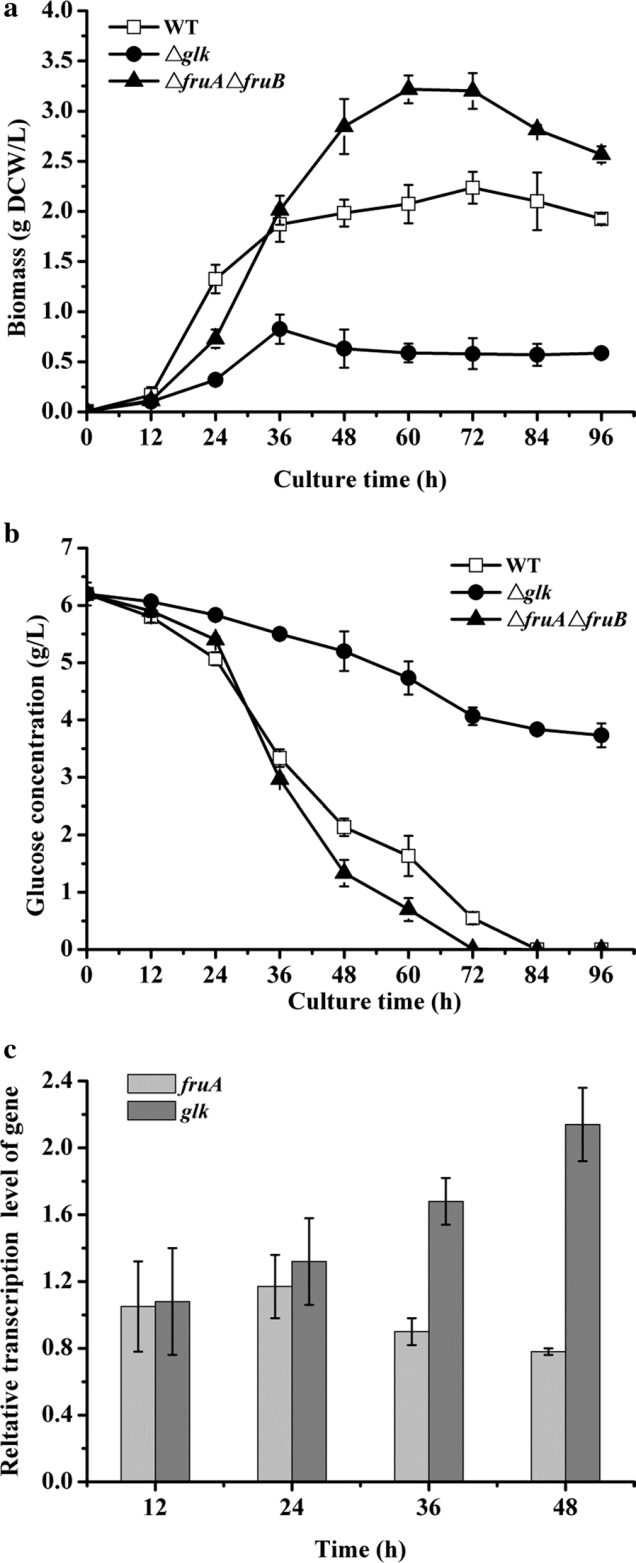
Growth and glucose metabolism of the WT and the mutant strains cultured in the MSMM, and RT-qPCR assay of the *fruA* and *glk* in the WT at different culture time. **a** Growth curves, **b** glucose concentration curves, and **c** relative transcription level

To clarify whether PTS (*fruAB*), non-PTS, or both of *R. sphaeroides* ATCC 17023 influences cellular glucose metabolism, two mutants (Δ*fruA*Δ*fruB* and Δ*glk*) were constructed using an in-frame markerless deletion method. The Δ*fruA*Δ*fruB* was a mutant with double knock out of *fruA* and *fruB*. Considering that no non-PTS type glucose transporter has been identified in *R. sphaeroides* presently, *glk* was knocked out to study the function of non-PTS in glucose metabolism. Subsequently, these mutants’ growth and glucose consumption were studied under aerobic incubation using glucose as the sole carbon source (Fig. 2). All mutant strains showed a lag phase at the beginning of cultivation (between 0 and 12 h), which was similar to that of the *R. sphaeroides* ATCC 17023 (WT) (Fig. 2a). Evident variations were observed among these strains since then. The WT went into an exponential growth phase and displayed a rapid growth rate than others between 12 and 24 h. Furthermore, the Δ*fruA*Δ*fruB* went into an exponential growth phase though the growth rate was slower than the WT; however, Δ*glk* still showed a slow growth rate. After that, the growth rate of the WT became slower from 36 to 72 h, going into a decline phase at 72 h. Δ*fruA*Δ*fruB* showed a faster growth rate between 24 and 36 h, and then the rate gradually slowed. The stationary growth phase was observed at approximately 60 h, and the decline phase appeared at 72 h for Δ*fruA*Δ*fruB*. In contrast, Δ*glk* continually kept a slow growth status between 24 and 36 h and went into a long stationary growth phase till the end of the experiment. Interestingly, the *Bio*_*max*_ obtained by Δ*fruA*Δ*fruB* was 3.22 ± 0.04 g DCW/L, which was much higher than that of the WT (*Bio*_*max*_ was 2.23 ± 0.07 g DCW/L) (Table 2). Although the Δ*glk* showed a typical bacterial growth process, both the growth rate and *Bio*_*max*_ were much weaker than the WT during the whole culture process that the *Bio*_*max*_ was 0.82 ± 0.01 g DCW/L achieved by the Δ*glk*.

**Table 2:** Growth and glucose consumption assay of the *R. sphaeroides* mutant strains and WT

Strain	Time (h)	Glucose metabolism (g/L)	*Bio*_max_^*^ (g DCW/L)	*r*_glc_^**^ (g/L/h)	*Y*_b/glc_^***^ (g/g)
WT	84^b^	6.21 ± 0.21^a^	2.23 ± 0.07^b^ (72 h)	0.074 ± 0.003^b^	0.36 ± 0.02^b^
Δ*glk*	96^a^	2.47 ± 0.04^b^	0.82 ± 0.01^c^ (96 h)	0.026 ± 0.001^c^	0.33 ± 0.01^c^
Δ*fruA*Δ*fruB*	72^c^	6.19 ± 0.16^a^	3.22 ± 0.04^a^ (60 h)	0.086 ± 0.002^a^	0.52 ± 0.03^a^

^*^Bio_*max*_ the maximum biomass; ^**^r_*glc*_ the average glucose consumption rate; ^***^Y_*b/glc*_ biomass yield to glucose consumption vs. the *Bio*_*max*_. Statistics analysis was performed based on one-way ANOVA method and the data in the same column with the same letters (a–c) meant no significant difference (P ≤ 0.05)

Additionally, glucose concentration was determined simultaneously (Fig. 2b). At the beginning of cultivation (0–12 h), the strains showed slow glucose consumption rates (*r*_*glc*_) that fit the characteristics of the lag phase. During the culture time between 12 and 24 h, the WT and Δ*fruA*Δ*fruB* sped up glucose consumption, though Δ*fruA*Δ*fruB* had a little slower consumption rate than the WT. The result could explain the reason why Δ*fruA*Δ*fruB* grew slower than the WT. Afterward, the residual glucose concentration in the group with Δ*fruA*Δ*fruB* was less than the WT. Finally, Δ*fruA*Δ*fruB* exhausted the glucose within 72 h, which was 12 h earlier than the WT. Compared with the WT, Δ*glk* showed a low ability on glucose consumption during the entire process. After incubation for 96 h, there was still 3.73 ± 0.21 g/L residual glucose in the medium. The *r*_*glc*_ of Δ*glk* was only 0.026 ± 0.001 g/L/h, whereas the *r*_*glc*_ of the Δ*fruA*Δ*fruB* could get to 0.086 ± 0.002 g/L/h, which was approximately 1.18 times that of the WT (Table 2). Besides the *r*_*glc*_, the *Y*_*b/glc*_ of the Δ*fruA*Δ*fruB* was also promoted, approximately 43.4% higher than that of WT. Additionally, we constructed the mutant strains, Δ*fruA* and Δ*fruB*. The results revealed that the two mutants showed similar growth and glucose metabolism status as those of the Δ*fruA*Δ*fruB* (unpublished data). Summarily, *glk* mutation seriously inhibited the growth and glucose metabolism of *R. sphaeroides*. Considering that *glk* is a vital gene involved in non-PTS, we speculated that the non-PTS played a major role in transporting glucose for *R. sphaeroides* ATCC 17023 during the entire process. However, deleting *fruAB* also influenced bacterial growth and glucose metabolism. The depressing effect of the *fruAB* mutation on growth appeared at the early incubation phase (12–24 h), whereas it showed a promotion effect on growth at the later phase (24–72 h). The influence of *fruAB* mutation on growth could be indirectly explained by the status of glucose metabolism. Therefore, we supposed that both the PTS and non-PTS had influence on glucose metabolism at the early culture phase. The relative transcriptional level of the *fruA* and *glk* in WT during cultivation was determined by RT-qPCR to verify the hypothesis further. The result was depicted in Fig. 2c, both *fruA* and *glk* showed an increasing tendency in the early culture phase (12–24 h). After that, the transcriptional level of *fruA* showed a decreased tendency since 36 h, whereas the *glk* still kept increasing from 36 to 48 h. Additionally, Δ*fruA*Δ*fruB*Δ*glk* was obtained through deleting the gene *glk* on the basis of Δ*fruA*Δ*fruB*. Comparing with Δ*glk*, both growth and glucose utilization were inhibited further at the early culture phase (Additional file 4: Fig. S4). To sum up, the results illustrate that both non-PTS and PTS have a function on glucose metabolism during the early phase, and the non-PTS played an important role in glucose metabolism.

### Enhancing the non-PTS pathway to promote cellular glucose metabolism

**Fig. 3:**
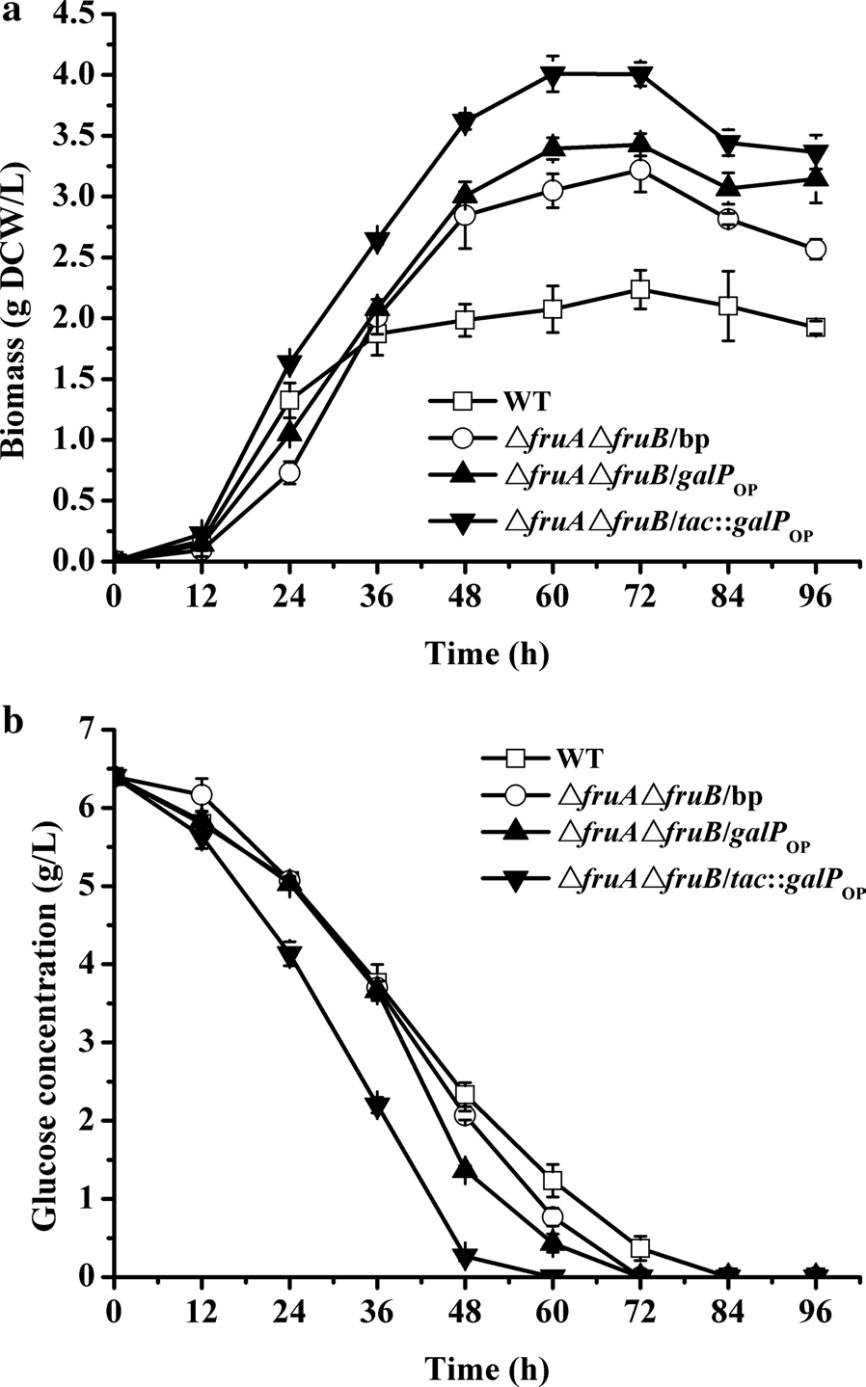
Growth and glucose metabolism of the WT and the mutant strains cultured in the MSMM. **a** Growth curves and **b** glucose concentration

According to the above study, blocking the non-PTS inhibited the glucose metabolism of *R. sphaeroides* ATCC 17023. Whether over-expressing the non-PTS-type glucose transporter helps in improving glucose catabolism. In this section, the galactose:H^+^ symporter (*galP*) from *E. coil* K-12 substr. MG1655 was selected for the study. First, three mutants, Δ*fruA*Δ*fruB*/bp, Δ*fruA*Δ*fruB*/*galP*_*OP*_, and Δ*fruA*Δ*fruB*/*tac*::*galP*_*OP*_*,* were constructed with the over-expression vectors, pBBR1MCS-2 and pBBR1MCS-2::*tac*. The Δ*fruA*Δ*fruB*/bp was directly introduced to the blank plasmid in Δ*fruA*Δ*fruB*. The Δ*fruA*Δ*fruB*/*galP*_*OP*_ was introduced to the plasmid, only harboring the gene, *galP*. The Δ*fruA*Δ*fruB*/*tac*::*galP*_*OP*_ is inserted with a strong promoter *tac* before the gene *galP* based on the pBBR1MCS-2::*tac*. Subsequently, these mutant strains were separately cultivated with glucose as the carbon source, and the biomass and glucose concentration was determined every 12 h. The Δ*fruA*Δ*fruB*/bp showed almost no difference from that of Δ*fruA*Δ*fruB* in growth and glucose metabolism (Fig. 3). This means that the plasmid introduction did not influence bacterial growth and glucose metabolism. Compared with Δ*fruA*Δ*fruB*/bp, the growth rate of Δ*fruA*Δ*fruB*/*galP*_*OP*_ was increased at the early phase (12–24 h), but the growth status was the same as that of Δ*fruA*Δ*fruB*/bp between 24 and 48 h (Fig. 3a). From 48 h, the biomass achieved by Δ*fruA*Δ*fruB*/*galP*_*OP*_ was higher than that of Δ*fruA*Δ*fruB/*bp though the growth trends were similar. The higher biomass achieved by Δ*fruA*Δ*fruB*/*galP*_*OP*_ could be explained by the faster glucose consumption rate than the Δ*fruA*Δ*fruB/*bp during this period. The result also suggested that the over-expression of *galP* could improve cellular glucose metabolism. For Δ*fruA*Δ*fruB*/*tac*::*galP*_*OP*_, the growth improved further than Δ*fruA*Δ*fruB*/*galP*_*OP*_ at the early phase (12–24 h), and then, it still kept a fast growth status than others until the time glucose was nearly exhausted. Additionally, the biomass quantity achieved was higher than that of Δ*fruA*Δ*fruB*/*galP*_*OP*_. The *Bio*_*max*_ was 4.01 ± 0.15 g DCW/L achieved by Δ*fruA*Δ*fruB*/*tac*::*galP*_*OP*_*,* which was the highest value among these strains. For glucose metabolism, Δ*fruA*Δ*fruB*/*tac*::*galP*_*OP*_ exhausted the glucose in the medium within 60 h, and the *r*_*glc*_ reached 0.107 ± 0.003 g/L/h (Table 3). Furthermore, *glk* was overexpressed in Δ*fruA*Δ*fruB* (Δ*fruA*Δ*fruB/tac*::*glk*)*.* However, both the growth and glucose metabolism decreased compared with Δ*fruA*Δ*fruB/*bp (Additional file 5: Fig. S5). Additionally, the result suggested that the original *glk* transcriptional level was fitting for glucose metabolism. Maybe, over-expression of the *glk* produced excessive glucose-6-P, which is toxic to cells.

**Table 3:** Growth and glucose metabolism of *R. sphaeroides* strains

Strain	Time (h)	Glucose metabolism (g/L)	*Bio*_max_^*^ (g DCW/L)	*r*_glc_^**^ (g/L/h)	*Y*_bio/glc_^***^ (g/g)
WT	84^b^	6.21 ± 0.21^a^	2.23 ± 0.07^d^ (72 h)	0.074 ± 0.003^c^	0.36 ± 0.02^b^
Δ*fruA*Δ*fruB/bp*	72^c^	6.18 ± 0.32^a^	3.21 ± 0.11^c^ (72 h)	0.086 ± 0.002^b^	0.52 ± 0.02^c^
Δ*fruA*Δ*fruB*/ *galP*_*OP*_	72^c^	6.21 ± 0.12^a^	3.43 ± 0.17^b^ (72 h)	0.086 ± 0.008^b^	0.55 ± 0.05^b^
Δ*fruA*Δ*fruB*/*tac*::*galP*_*OP*_	60^a^	6.20 ± 0.17^a^	4.01 ± 0.15^a^ (60 h)	0.103 ± 0.003^a^	0.65 ± 0.07^a^

^*^Bio_*max*_ the maximum biomass; ^**^r_*glc*_ the average glucose consumption rate; ^***^Y_*bio/glc*_ biomass yield to glucose consumption versus the *Bio*_*max*_. Statistics analysis was performed based on one-way ANOVA method and the data in the same column with the same letters (a–d) meant no significant difference (P ≤ 0.05)

### Improving CoQ_10_ productivity of ***R. sphaeroides***

The CoQ_10_ content of these mutants was determined, and the result is presented in Table 4. Compared with the WT, Δ*glk*, Δ*fruA*Δ*fruB*, and Δ*fruA*Δ*fruB*/*tac*::*galP*_*OP*_ synthesized a low content of CoQ_10_ when incubated for 24 h; especially, the CoQ_10_ content of Δ*glk* was 3.12 ± 0.04 mg/g DCW. After that, CoQ_10_ content of Δ*fruA*Δ*fruB* and Δ*fruA*Δ*fruB*/*tac*::*galP*_*OP*_ was increased after 48 h, whereas the CoQ_10_ content of the WT and Δ*glk* showed a slight reduction. As incubation proceeded (48–96 h), the CoQ_10_ content of the WT and Δ*glk* stopped reducing and increased. Simultaneously, Δ*fruA*Δ*fruB* and Δ*fruA*Δ*fruB*/*tac*::*galP*_*OP*_ increased in the CoQ_10_ content. Finally, the CoQ_10_ content of Δ*fruA*Δ*fruB* and Δ*fruA*Δ*fruB*/*tac*::*galP*_*OP*_ reached 5.02 ± 0.18 and 5.11 ± 0.14 mg/g DCW, respectively. The maximum CoQ_10_ content of Δ*fruA*Δ*fruB*/*tac*::*galP*_*OP*_ was increased by 29.4% than the WT. It can be proposed that the mutation of *fruAB* improved *Y*_*b/glc*_ and bacterial *r*_*glc*_ but also enhanced the CoQ_10_ synthesis of *R. sphaeroides*. Moreover, strengthening glucose transportation by over-expressing *galP* showed little help to strengthen CoQ_10_ synthesis.

**Table 4:** The CoQ_10_ content of WT and mutants cultured in SMM

Culture time (h)	CoQ_10_ content (mg/g DCW)			
	WT	Δ*glk*	Δ*fruA*Δ*fruB*	Δ*fruA*Δ*fruB*/*tac*::*galP*_*OP*_
24	3.79 ± 0.11^a^	3.12 ± 0.04^d^	3.23 ± 0.17^c^	3.43 ± 0.16^b^
48	3.62 ± 0.06^b^	2.53 ± 0.33^c^	3.65 ± 0.05^b^	3.76 ± 0.21^a^
72	3.83 ± 0.13^b^	2.85 ± 0.05^c^	4.97 ± 0.15^a^	5.01 ± 0.33^a^
96	3.95 ± 0.21^c^	3.02 ± 0.19^d^	5.02 ± 0.18^b^	5.11 ± 0.14^a^

Statistical analysis was performed based on one-way ANOVA and the data with the same letters (a–e) means no significant difference (P ≤ 0.05) for each line

**Fig. 4:**
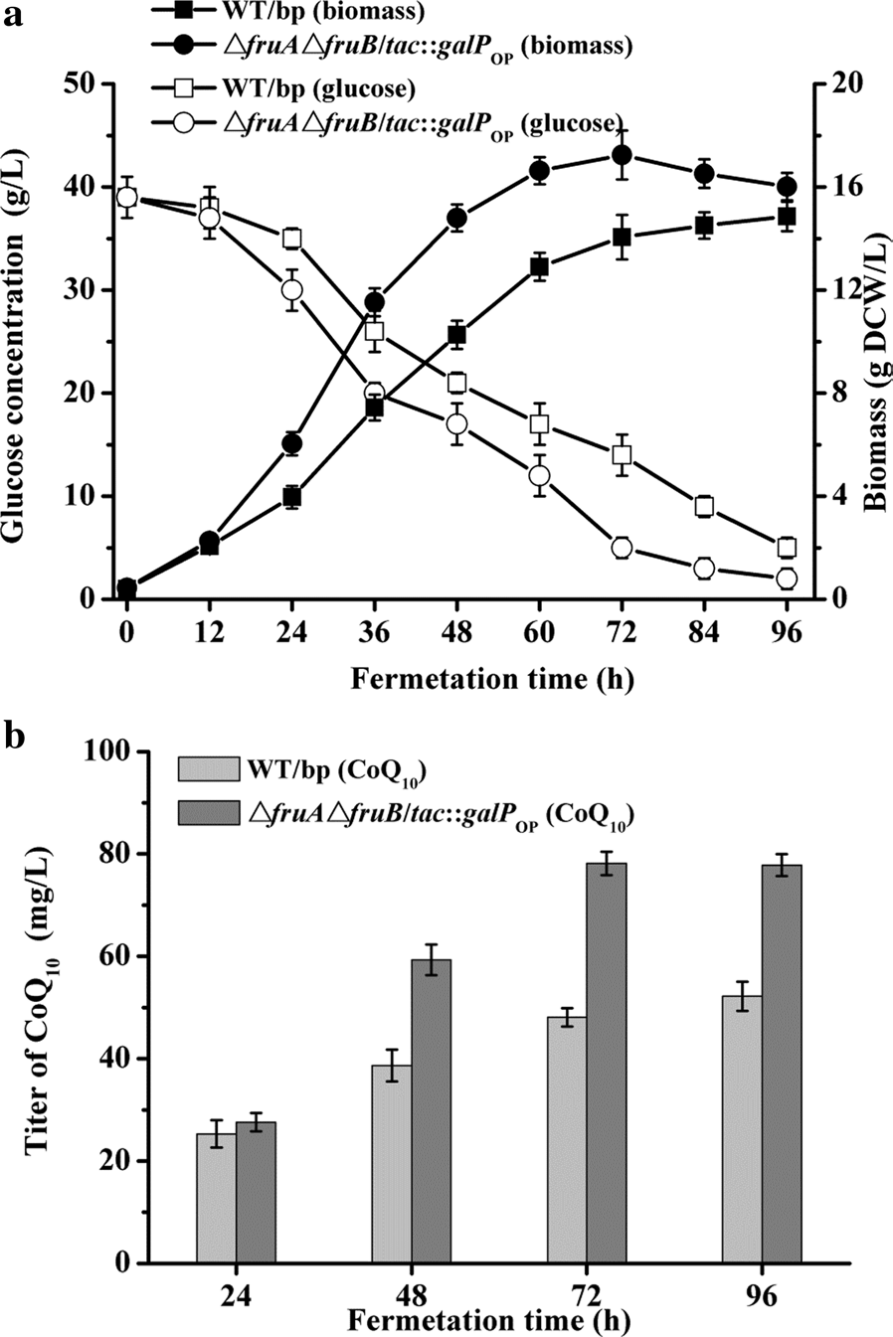
Comparison of the growth, glucose metabolism and titer of CoQ_10_ of the WT and the Δ*fruA*Δ*fruB*/*tac*::*galP*_*OP*_ cultured in the fermentation medium B. **a** Growth and glucose concentration curves, and **b** Titer of CoQ_10_ during fermentation

Although *galP* over-expression in Δ*fruA*Δ*fruB* played no role in promoting the CoQ_10_ synthesis ability of *R. sphaeroides*, the strategy can promote glucose metabolism rate, which shortens the fermentation time. The inactivation of *fruAB* improved *Y*_*b/glc*_ and the bacterial CoQ_10_ synthetic ability. Considering the advantages of the two strategies, Δ*fruA*Δ*fruB*/*tac*::*galP*_*OP*_ was applied to CoQ_10_ fermentation in a lab-scale tank (1 L), evaluating whether the CoQ_10_ fermentation is improved. Δ*fruA*Δ*fruB*/*tac*::*galP*_*OP*_ showed an evident improvement in growth compared with the WT/bp during the fermentation process (12–72 h) (Fig. 4a). The *Bio*_*max*_ of Δ*fruA*Δ*fruB*/*tac*::*galP*_*OP*_ was harvested at 72 h of fermentation, which was 24 h earlier than the WT/bp. Moreover, the value of the *Bio*_*max*_ reached 17.24 ± 0.97 g DCW/L, which was promoted by approximately 16% higher than that of the WT/bp (14.85 ± 0.57 g DCW/L). Simultaneously, the glucose concentration in the medium was almost exhausted after 72 h for Δ*fruA*Δ*fruB*/*tac*::*galP*_*OP*_ (< 5 g/L), whereas there was more than 10-g/L residual glucose residual for the WT/bp. In the aspect of CoQ_10_ synthesis (Fig. 4b), the yield gradually increased as the fermentation proceeded for both strains. At 48 h incubation, the yield of Δ*fruA*Δ*fruB*/*tac*::*galP*_*OP*_ showed a higher level than that of the WT/bp, and the phenomenon lasted to the end. The maximum CoQ_10_ titer of Δ*fruA*Δ*fruB*/*tac*::*galP*_*OP*_ reached 78.14 ± 2.31 mg/L, which was approximately 49.76% higher than that of the WT/bp. Moreover, Δ*fruA*Δ*fruB*/*tac*::*galP*_*OP*_ achieved the maximum CoQ_10_ titer at 72 h, which was 24 h earlier than the WT/bp.

## Discussion

For many bacteria, PTS^Glc^ is the first-selected pathway to transport exogenous glucose [6, 22]. After that, some other sugar-specific-PTSs, such as the PTS^Fru^ (*fruAB*), have been identified with the same function as that of the PTS^Glc^ [6, 23]. In *R. sphaeroides* ATCC 17023, no PTS^Glc^-encoding genes are identified presently, but it has a PTS^Fru^ encoding gene cluster (*fruAB*). It is revealed that glucose metabolism and bacterial growth were influenced by the mutation of the *fruAB* in *R. sphaeroides* ATCC 17023 (Fig. 2). Interestingly, the result of *fruAB* mutation revealed two effects on bacterial growth during the whole culture process. It showed an inhibition effect at the early cultivation phase (12–24 h) and displayed a promoting effect at the late phase at 36 h. Finally, the *Bio*_*max*_ received by Δ*fruA*Δ*fruB* was much higher than that of the WT. The glucose metabolism of Δ*fruA*Δ*fruB* also displayed a fit change characteristic vs. that of the growth change. Additionally, RT-qPCR assay revealed that the transcription level of the *fruA* in the WT kept a relatively high level at the early cultivation phase (12–24 h) and then decreased at 36 h. With the comprehensive analysis of the results, we supposed that the PTS^Fru^ in *R. sphaeroides* ATCC 17023 majorly joined in the glucose metabolism at the early cultivation phase. The PTS^Glc^ is considered an effective way to utilize glucose because only one PEP is coupled with the translocation-phosphorylation of PTS carbohydrates when forming one ATP [6, 22]. In contrast, glucose utilization through a non-PTS transporter requires extra ATP to phosphate a carbohydrate molecule in the carbohydrate kinase reaction. Regarding energy consumption, bacteria synthesize cellular skeleton materials at the early cultivation phase, requiring a large amount of energy; thus, the PTS type system may be a good choice for saving energy at the early growth phase. As cultivation continued, the function of *fruAB* was weakened. The result might be due to the energy production ability of the bacteria, which is not as a limiting factor for cell growth after the lag phase. The high biomass achieved by Δ*fruA*Δ*fruB* meant that more carbon fluxed to cellular assimilation metabolism. For the native glucose utilization pathway in *E. coli*, half of the PEP produced is used for glucose uptake and phosphorylation. PEP is an essential precursor for synthesizing many chemicals, such as succinate, malate, and aromatic compounds. In this sense, *fruAB* mutation might reduce PEP catabolism, which helps to synthesize cytoskeleton substances after the lag growth phase. The similar phenomenon is also found in the mutation of PTS^Ntr^ in *P. putida*, which is due to the enhancement of anabolism [16]. The phenomenon obtained from *fruAB* mutation is interesting though the mechanism is unclear. In future studies, more efforts will be put on disclosing the mechanism for promoting growth and its use.

The non-PTS composes of sugar transporters and glucokinase (*glk*). Glucose transports into cells by non-PTS in a non-phosphorylated form and then phosphorylated by the glucokinase for subsequent metabolism. A *glk* gene exists in the genome of *R. sphaeroides* ATCC 17023. The result revealed that the mutation of *glk* decreased bacterial growth when cultured in the medium with glucose as the sole carbon source. In addition, a poor glucose consumption status was observed for the mutant Δ*glk*. RT-qPCR assay revealed that the transcriptional level of *glk* in WT kept an increased tendency as the cultivation continued. The above results revealed that the non-PTS played an important role in controlling glucose metabolism of *R. sphaeroides* ATCC 17023 during the culture process, especially since exponential growth phase, though the corresponding sugar-specific transporters were still unidentified. The result agrees with other *R. sphaeroides* strains [18].

Galactose permease (*galP*) is a galactose:H^+^ symporter belonging to the MFS [8]. It was reported that the *E. coli* PTS^−^glucose^+^ strain could transport glucose by a non-PTS mechanism as fast as its WT parental strain [23]. Further research showed that the *gal* regulon genes, which encode non-PTS transporter and enzymes for galactose metabolism, are enhanced in this mutant; furthermore, rapid glucose consumption depends on the low-affinity GalP. However, the over-expression of a heterogeneous *galP* in Δ*fruA*Δ*fruB* improved the decrease in growth generated by the *fruAB* mutation at the early cultivation phase (Fig. 3). Alternatively, it could enhance glucose metabolism and promote biomass accumulation during the entire cultivation process. The results further illustrated that *R. sphaeroides* ATCC 17023 glucose metabolism mainly relied on the non-PTS. Additionally, it suggested the glucose transmembrane was an important limitation for the glucose metabolism of this bacterium. Furthermore, over-expressing *glk* was harmful to bacterial growth (Additional file 5: Fig. S5); bacterial glucose metabolism was also inhibited. Excessive glucose-6-p might be accumulated in the cytoplasm because of the over-expression of *glk*, which has toxic effect on bacterial metabolism. Finding an appropriate expression level of *glk* may solve the question.

Mutation of the *fruAB* influenced glucose metabolism and mediated the synthesis of CoQ_10_ in *R. sphaeroides* ATCC 17023. Presently, the mechanism of *fruAB* mutation on increasing bacterial CoQ_10_ synthesis is unknown. PEP is an important precursor for synthesizing aromatic compounds by the shikimate pathway. Aromatic compounds are vital sources of the benzene ring of CoQ_10_. Additionally, we previously knocked out the pyruvate kinase (*pykA*) of *R. sphaeroides* ATCC 17023, transforming PEP to pyruvate. The mutant showed higher CoQ_10_ content than that of the WT (unpublished data). Considering the relationship between PEP and CoQ_10_, we supposed that PTS^Fru^ inactivation might reduce the catabolism quantity of PEP, which was promoted more PEP flow to CoQ_10_ synthesis. However, the CoQ_10_ content of Δ*fruA*Δ*fruB*/*tac*::*galP*_*OP*_ showed no evident increase compared with that of Δ*fruA*Δ*fruB*. The result suggested that only enhancing glucose transport cannot promote bacterial CoQ_10_ synthesis ability.

## Conclusion

The two sugar transmembrane pathways in *R. sphaeroides* ATCC 17023 influenced growth and glucose metabolism. The PTS^Fru^ mutation revealed two effects on bacterial growth: inhibition at the early cultivation phase and promotion later. *glk* mutation decreased growth and glucose metabolism. Additionally, compared with the non-PTS, PTS^Fru^ had a relationship with CoQ_10_ synthesis that destroying the PTS^Fru^ could enhance bacterial CoQ_10_ synthesis ability. Enhancing glucose transport of the non-PTS with over-expressing a galactose:H^+^ symporter (*galP*) in Δ*fruA*Δ*fruB* relieved the inhibition effect and enhanced growth. Moreover, the over-expression of *galP* has little effect on enhancing bacterial CoQ_10_ synthesis ability. According to the functional study of *fruAB* and *glk*, CoQ_10_ fermentation was improved through several modifications in glucose metabolism (constructed the Δ*fruA*Δ*fruB*/*tac*::*galP*_*OP*_) and was verified as available for fermentation in 1 L bioreactors. Summarily, our study provided a new guidance for improving CoQ_10_ productivity of *R. sphaeroides*.

## Supplementary Information

**Additional file 1:** **Fig. S1** **(a) **Construction flowchart of the *glk* gene deletion vector pK18*mobsacB*::*glk*-L-R; **(b)** Construction of *glk* gene deletion vector pK18*mobsacB*::*glk*-L-R; and **(c)** Filtration and verification of Δ*glk*.

**Additional file 2:** **Fig. S2** **(a) **Construction flowchart of the *fruB* gene deletion vector pK18*mobsacB*::*fruB*-L-R; **(b)** Construction of *glk* gene deletion vector pK18*mobsacB*::* fruA*-L-R;** (c)** Construction of *glk* gene deletion vector pK18*mobsacB*::* fruB* -L-R; **(d)** Filtration and verification of Δ*fruA*; and **(e)** Filtration and verification of Δ*fruA*Δ*fruB*.

**Additional file 3:** **Fig. S3** **(a) **Construction flowchart of the *galP* gene overexpression vector pBBR1MCS-2::*tac*:;*galP*; **(b)** Construction of he *galP* gene overexpression vector pBBR1MCS-2::*tac*:;*galP*; and **(c)** Filtration and verification of the Δ*fruA*Δ*fruB*/*tac*::*galP*_*OP*_.

**Additional file 4:** **Fig. S4 **Growth and glucose metabolism of the WT and the mutant strains cultured in the MSMM. (a) Growth curves and (b) glucose concentration curves.

**Additional file 5:** **Fig. S5 **Growth and glucose metabolism of the Δ*fruA*Δ*fruB/*bp and the Δ*fruA*Δ*fruB/tac*::*glk* cultured in the MSMM.

**Additional file 6: Table S1** Primers and restriction enzymes used in this study.

**Additional file 7: Table S2** Primers used for mutant strains verification.

**Additional file 8**: **Table S3** Primers used for RT-qPCR amplification.

## References

[1] Li F, Zhao Y, Li B, Qiao J, Zhao G (2016). Engineering *Escherichia coli* for production of 4-hydroxymandelic acid using glucose-xylose mixture. Microb Cell Fact.

[2] Tong X, Oh EK, Lee BH, Lee JK. Production of long-chain free fatty acids from metabolically engineered *Rhodobacter sphaeroides* heterologously producing periplasmic phospholipase A2 in dodecane-overlaid two-phase culture. Microbial Cell Fact. 2019;18. 10.1186/s12934-019-1070-8 PMC6357386 30704481

[3] Wang S, Fang Y, Wang Z, Zhang S, Wang X (2021). Improving l-threonine production in *Escherichia coli* by elimination of transporters ProP and ProVWX. Microb Cell Fact.

[4] Wurm DJ, Veiter L, Ulonska S, Eggenreich B, Herwig C, Spadiut O (2016). The *E. coli* pET expression system revisited-mechanistic correlation between glucose and lactose uptake. Appl Microbiol Biotechnol.

[5] Poblete-Castro I, Binger D, Rodrigues A, Becker J, Martins dos Santos VAP, Wittmann C (2013). In-silico-driven metabolic engineering of *Pseudomonas putida* for enhanced production of poly-hydroxyalkanoates. Metabol Eng.

[6] Luo Y, Zhang T, Wu H (2014). The transport and mediation mechanisms of the common sugars in *Escherichia coli*. Biotechnol Adv.

[7] Wurm DJ, Hausjell J, Ulonska S, Herwig C, Spadiut O (2017). Mechanistic platform knowledge of concomitant sugar uptake in *Escherichia coli* BL21(DE3) strains. Sci Rep.

[8] Fordjour E, Adipah FK, Zhou S, Du G, Zhou J (2019). Metabolic engineering of *Escherichia coli* BL21 (DE3) for de novo production of l-DOPA from d-glucose. Microb Cell Fact.

[9] Chavarría M, Kleijn RJ, Sauer U, Pflüger-Grau K, Lorenzo VD (2012). Regulatory tasks of the phosphoenolpyruvate-phosphotransferase system of *Pseudomonas putida* in central carbon metabolism. MBio.

[10] Lu J, Tang J, Liu Y, Zhu X, Zhang T, Zhang X (2012). Combinatorial modulation of *galP* and *glk* gene expression for improved alternative glucose utilization. Appl Microbiol Biotechnol.

[11] Ikeda M (2012). Sugar transport systems in *Corynebacterium glutamicum*: features and applications to strain development. Appl Microbiol Biotechnol.

[12] Gonzy-Tréboul G, Zagorec M, Rain-Guion MC, Steinmetz M (2010). Phosphoenolpyruvate:sugar phosphotransferase system of *Bacillus subtilis*: nucleotide sequence of *ptsX*, *ptsH* and the 5′-end of* ptsI* and evidence for a *ptsHI* operon. Mol Microbiol.

[13] Su A, Chi S, Li Y, Tan S, Qiang S, Chen Z, Meng Y (2018). Metabolic redesign of *Rhodobacter sphaeroides* for lycopene production. J Agric Food Chem.

[14] Zhang J, Gao D, Cai J, Liu H, Qi Z (2018). Improving coenzyme Q_10_ yield of *Rhodobacter sphaeroides* via modifying redox respiration chain. Biochem Eng J.

[15] Chen X, Jiang X, Xu M, Zhang M, Huang R, Huang J, Qi F (2019). Co-production of farnesol and coenzyme Q_10_ from metabolically engineered *Rhodobacter sphaeroides*. Microb Cell Fact.

[16] Lee S, Tan TS, Kawamukai M, Chen ES (2017). Cellular factories for coenzyme Q_10_ production. Microb Cell Fact.

[17] Raizner AE, Quiones MA (2021). Coenzyme Q_10_ for patients with cardiovascular disease. J Am Coll Cardiol.

[18] Imam S, Noguera DR, Donohue TJ (2015). CceR and AkgR regulate central carbon and energy metabolism in Alphaproteobacteria. MBio.

[19] Fuhrer T, Fischer E, Sauer U (2005). Experimental identification and quantification of glucose metabolism in seven bacterial species. J Bacteriol.

[20] Luo Y, Ge M, Wang B, Sun C, Wang J, Dong Y, Xi J (2021). CRISPR/Cas9-deaminase enables robust base editing in *Rhodobacter sphaeroides* 2.4.1. Microbial Cell Fact.

[21] Szymona M, Doudoroff M (1960). Carbohydrate metabolism in *Rhodopseudomonas sphreoides*. J Gen Microbiol.

[22] Kyselova L, Kreitmayer D, Kremling A, Bettenbrock K (2018). Type and capacity of glucose transport influences succinate yield in two-stage cultivations. Microb Cell Fact.

[23] Saier MH (2000). Families of transmembrane sugar transport proteins. Mol Microbiol.

