# Supplementary material for: Improving CoQ10 productivity by strengthening glucose transmembrane of Rhodobacter sphaeroides
Source: Microb Cell Fact. 2021 Oct 30;20:207. doi: 10.1186/s12934-021-01695-z (PMC8557541; doi:10.1186/s12934-021-01695-z)
Supplement: Supplementary file 1 — Additional file 1: Fig. S1 (a) Construction flowchart of the glk gene deletion vector pK18mobsacB::glk-L-R; (b) Construction of glk gene deletion vector pK18mobsacB::glk-L-R; and (c) Filtration and verification of Δglk. [file 12934_2021_1695_MOESM1_ESM.docx]

**Fig.S1**

**(a)**


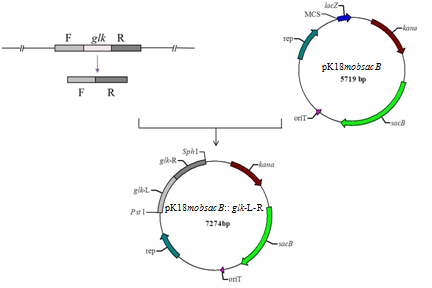


**(b)**


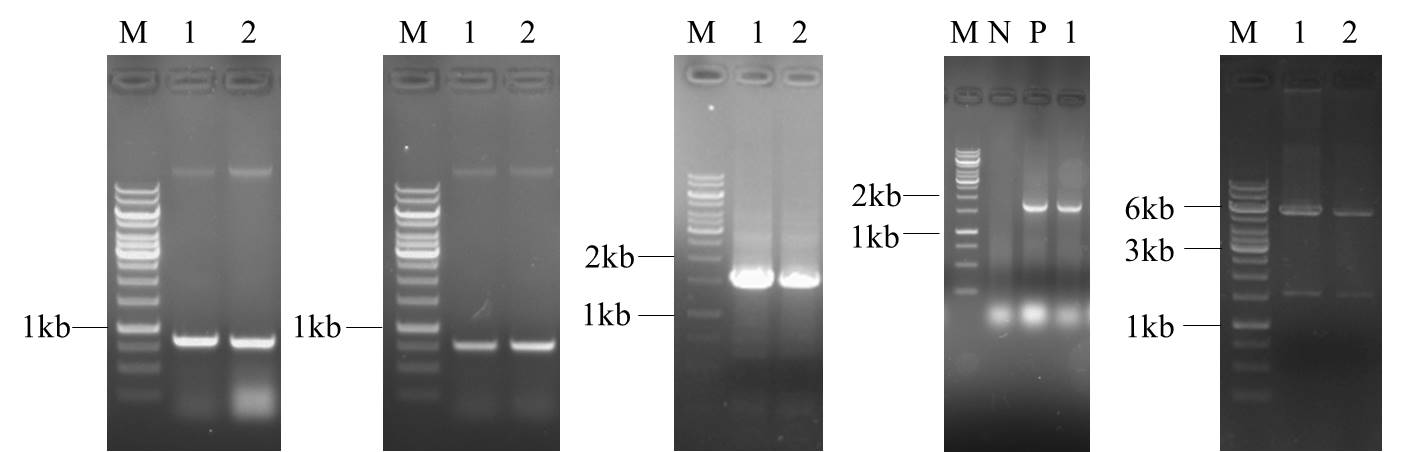


**(c)**


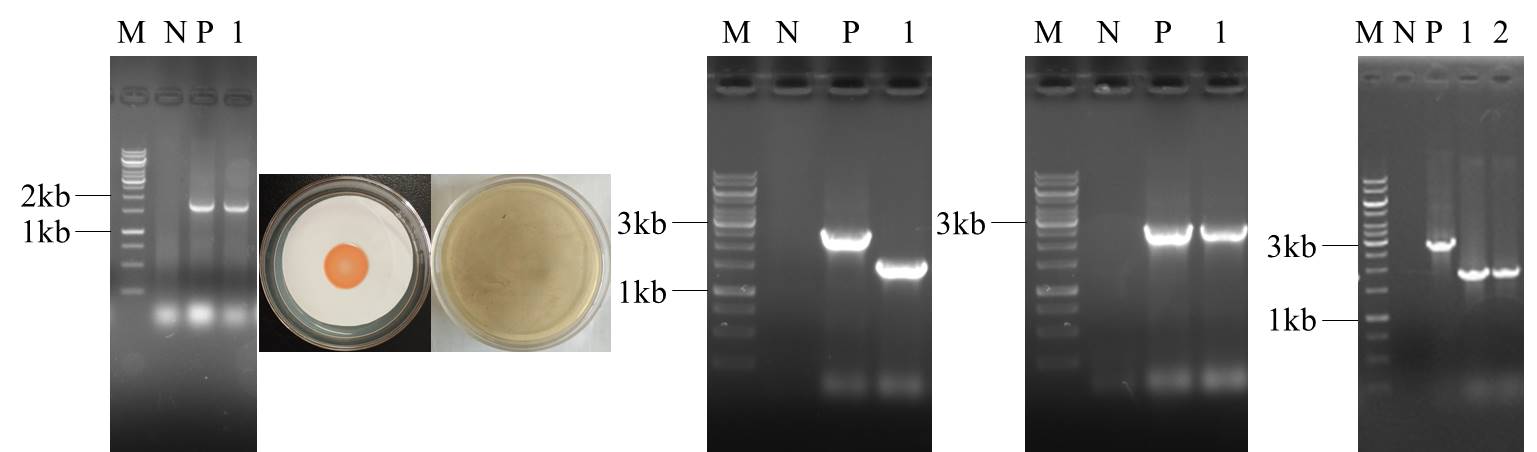


**Fig.S1** **(a)** Construction flowchart of the *glk* gene deletion vector pK18*mobsacB*::*glk*-L-R; **(b)** Construction of *glk* gene deletion vector pK18*mobsacB*::*glk*-L-R; and **(c)** Filtration and verification of △*glk*.
