# Supplementary material for: Improving CoQ10 productivity by strengthening glucose transmembrane of Rhodobacter sphaeroides
Source: Microb Cell Fact. 2021 Oct 30;20:207. doi: 10.1186/s12934-021-01695-z (PMC8557541; doi:10.1186/s12934-021-01695-z)
Supplement: Supplementary file 2 — Additional file 2: Fig. S2 (a) Construction flowchart of the fruB gene deletion vector pK18mobsacB::fruB-L-R; (b) Construction of glk gene deletion vector pK18mobsacB:: fruA-L-R; (c) Construction of glk gene deletion vector pK18mobsacB:: fruB -L-R; (d) Filtration and verification of ΔfruA; and (e) Filtration and verification of ΔfruAΔfruB. [file 12934_2021_1695_MOESM2_ESM.docx]

**Fig.S2**

**(a)**


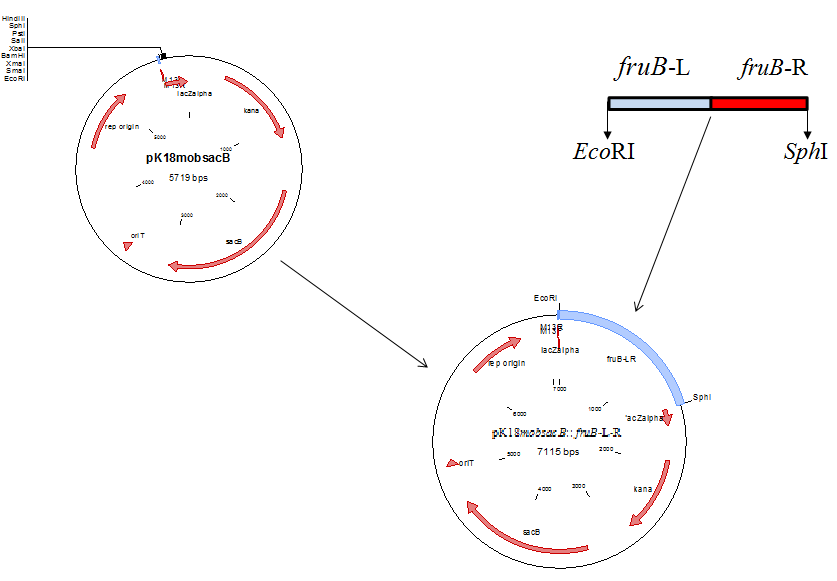


**(b)**


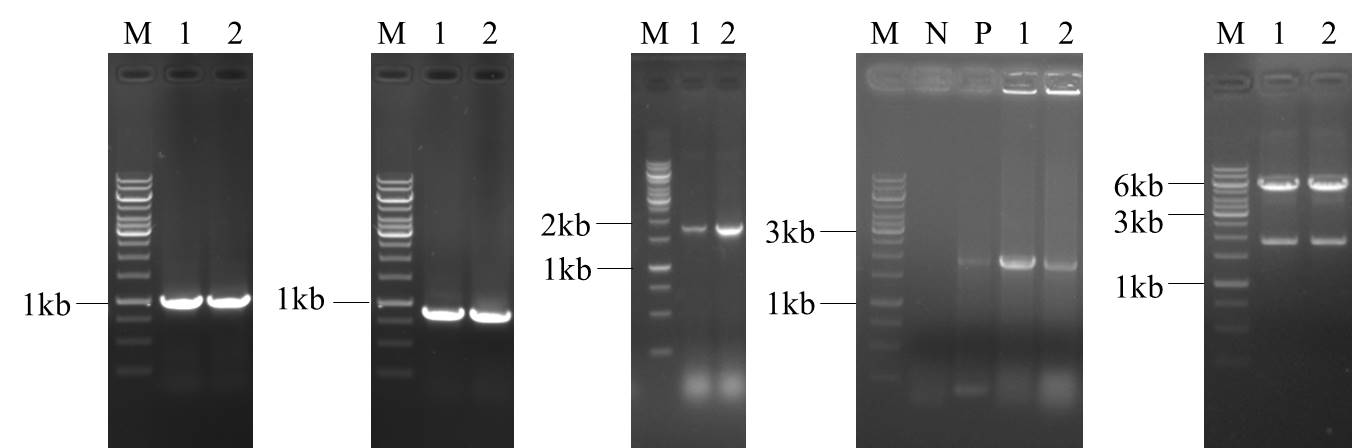


**(c)**


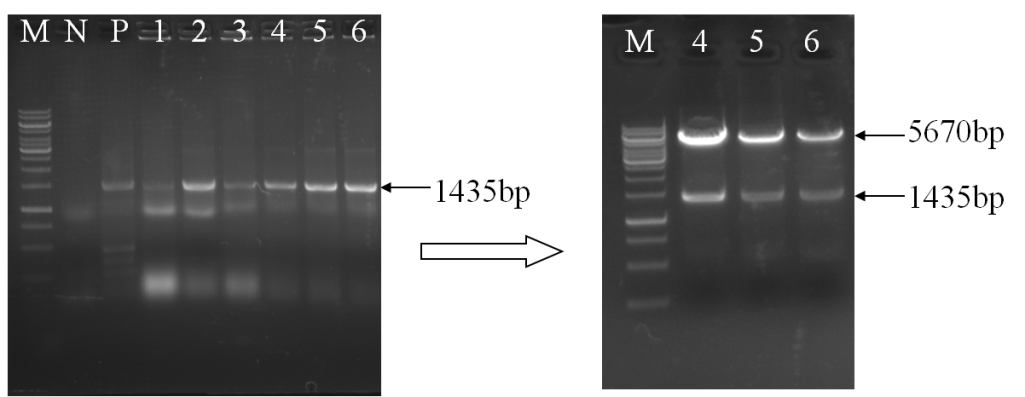


**(d)**


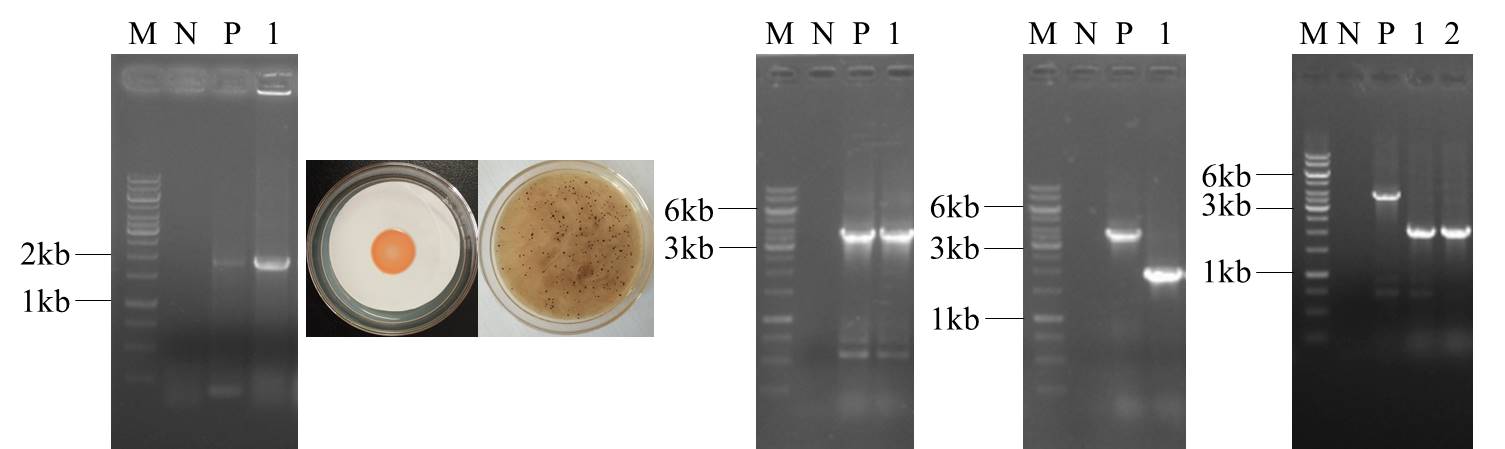


**(e)**


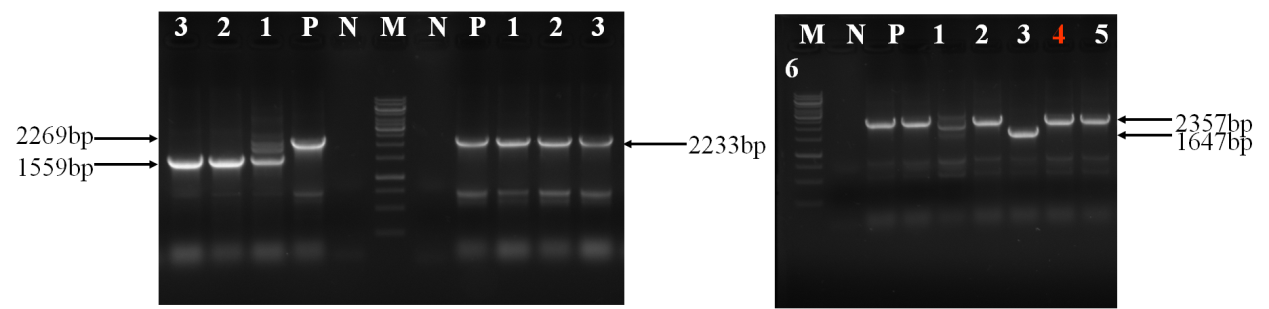


**Fig.S2** **(a)** Construction flowchart of the *fruB* gene deletion vector pK18*mobsacB*::*fruB*-L-R; **(b)** Construction of *glk* gene deletion vector pK18*mobsacB*:: *fruA*-L-R; **(c)** Construction of *glk* gene deletion vector pK18*mobsacB*:: *fruB* -L-R; **(d)** Filtration and verification of △*fruA*; and **(e)** Filtration and verification of △*fruA*△*fruB*.
