# Supplementary material for: Improving CoQ10 productivity by strengthening glucose transmembrane of Rhodobacter sphaeroides
Source: Microb Cell Fact. 2021 Oct 30;20:207. doi: 10.1186/s12934-021-01695-z (PMC8557541; doi:10.1186/s12934-021-01695-z)
Supplement: Supplementary file 3 — Additional file 3: Fig. S3 (a) Construction flowchart of the galP gene overexpression vector pBBR1MCS-2::tac:;galP; (b) Construction of he galP gene overexpression vector pBBR1MCS-2::tac:;galP; and (c) Filtration and verification of the ΔfruAΔfruB/tac::galPOP. [file 12934_2021_1695_MOESM3_ESM.docx]

**Fig. S3**

**(a)**

**
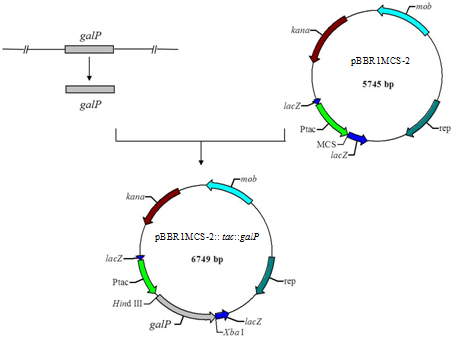
**

**(b)**


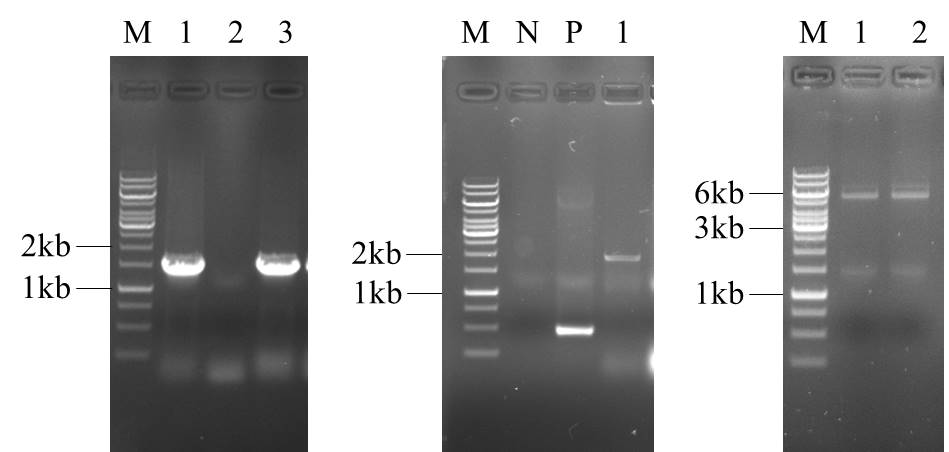


**(c)**


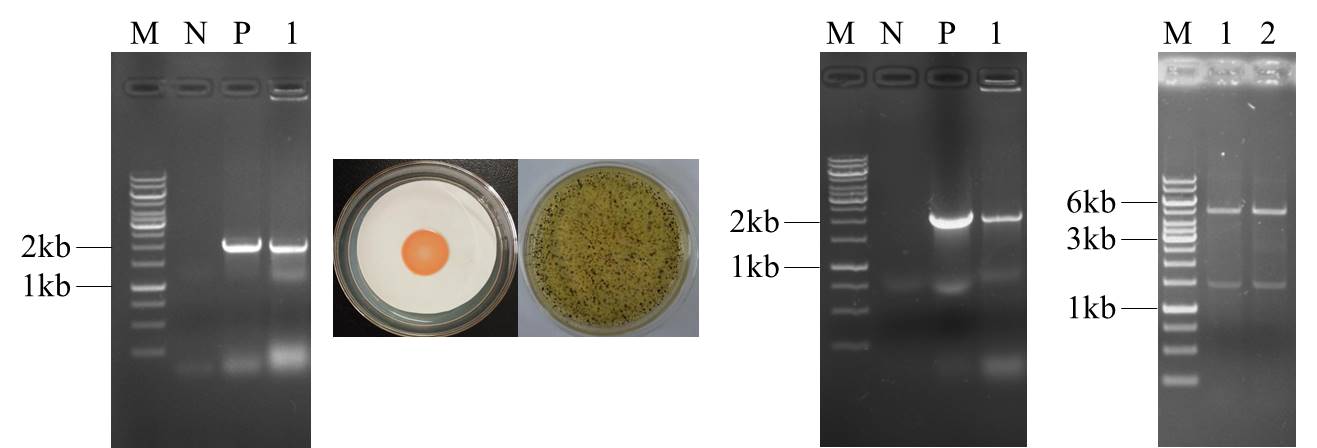


**Fig.S3** **(a)** Construction flowchart of the *galP* gene overexpression vector pBBR1MCS-2::*tac*:;*galP*; **(b)** Construction of he *galP* gene overexpression vector pBBR1MCS-2::*tac*:;*galP*; and **(c)** Filtration and verification of the △*fruA*△*fruB*/*tac*::*galP_OP_* .
