# Supplementary material for: Improving CoQ10 productivity by strengthening glucose transmembrane of Rhodobacter sphaeroides
Source: Microb Cell Fact. 2021 Oct 30;20:207. doi: 10.1186/s12934-021-01695-z (PMC8557541; doi:10.1186/s12934-021-01695-z)
Supplement: Supplementary file 4 — Additional file 4: Fig. S4 Growth and glucose metabolism of the WT and the mutant strains cultured in the MSMM. (a) Growth curves and (b) glucose concentration curves. [file 12934_2021_1695_MOESM4_ESM.docx]

**Fig.S4**

**(a)**





**(b)**

**

**

**Fig.S4** Growth and glucose metabolism of the WT and the mutant strains cultured in the MSMM. (a) Growth curves and (b) glucose concentration curves.
