# Supplementary material for: Improving CoQ10 productivity by strengthening glucose transmembrane of Rhodobacter sphaeroides
Source: Microb Cell Fact. 2021 Oct 30;20:207. doi: 10.1186/s12934-021-01695-z (PMC8557541; doi:10.1186/s12934-021-01695-z)
Supplement: Supplementary file 5 — Additional file 5: Fig. S5 Growth and glucose metabolism of the ΔfruAΔfruB/bp and the ΔfruAΔfruB/tac::glk cultured in the MSMM. [file 12934_2021_1695_MOESM5_ESM.docx]

**Fig.S5**


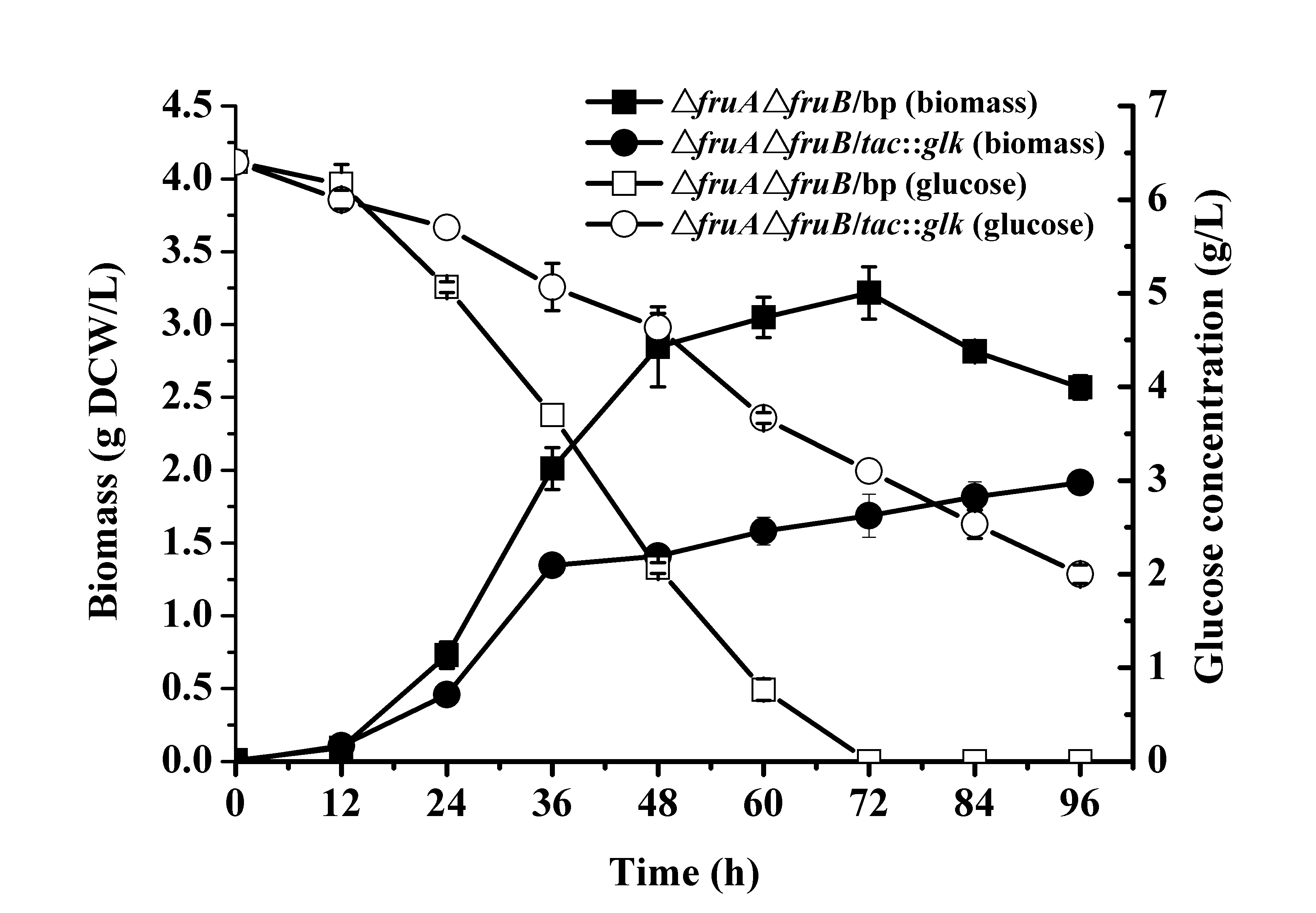


**Fig.S5** Growth and glucose metabolism of the △*fruA*△*fruB/*bp and the △*fruA*△*fruB/tac*::*glk* cultured in the MSMM.
