# Supplementary material for: Improving CoQ10 productivity by strengthening glucose transmembrane of Rhodobacter sphaeroides
Source: Microb Cell Fact. 2021 Oct 30;20:207. doi: 10.1186/s12934-021-01695-z (PMC8557541; doi:10.1186/s12934-021-01695-z)
Supplement: Supplementary file 6 — Additional file 6: Table S1 Primers and restriction enzymes used in this study. [file 12934_2021_1695_MOESM6_ESM.doc]

**Table S1** Primers and restriction enzymes used in this study

| Primers | Sequences (5’-3’) | Underlined sequence |
| --- | --- | --- |
| *glk*-L-F | AACTGCAGTCGCACTTCCTCGGCCATCA | *Pst* I |
| *glk*-L-R | TGCCGCCCGAGAGCGGGGCGTTCTCCGGACTGGCTTA |  |
| *glk*-R-F | TAAGCCAGTCCGGAGAACGCCCCGCTCTCGGGCGGCA |  |
| *glk*-R-R | ACATGCATGCCGAGGCGAAGGCGCATTATC | *Sph* I |
| *fruA*-L-F | ACGCGTCGACTCCATACCGTCACGCTCAACCC | *Sal* I |
| *fruA*-L-R | GTCAGACGGCCGAGGGCACCTGTCATGTCCTCTCTCCCTG |  |
| *fruA*-R-F | CAGGGAGAGAGGACATGACAGGTGCCCTCGGCCGTCTGAC |  |
| *fruA*-R-R | CCGGAATTCAGGTGCCGACGATCCGCTA | *Eco*R I |
| *fruB*-L-F | CCGGAATTCGCCTCGCCGGTGAAGGAGTT | *Eco*R I |
| *fruB*-L-R | CGGTATGGACGCGGCTCATG CGCGTCCAGTTGGATCAGTTCG |  |
| *fruB*-R-F | CGAACTGATCCAACTGGACGCGCATGAGCCGCGTCCATACCG |  |
| *fruB*-R-R | ACATGCATGCCCGTCCGACGAGACGAAGAGC | *Sph* I |
| *galP_op_-*F | GCTCTAGATTACGTCGTACTCACCTATC | *Xba* I |
| *galP_op_-*R | CGAGCTCAAGATGACTGCAAGAGGTGG | *Sac* I |
| *glk_op_-*F | CCCAAGCTTAGCCAGTCCGGAGAACGCCAT | *Hin*d III |
| *glk_op_-*R | GCTCTAGAAGGTTTGGCGGTCTGCGTTA | *Xba* I |
