# Supplementary material for: Improving CoQ10 productivity by strengthening glucose transmembrane of Rhodobacter sphaeroides
Source: Microb Cell Fact. 2021 Oct 30;20:207. doi: 10.1186/s12934-021-01695-z (PMC8557541; doi:10.1186/s12934-021-01695-z)
Supplement: Supplementary file 7 — Additional file 7: Table S2 Primers used for mutant strains verification. [file 12934_2021_1695_MOESM7_ESM.docx]

**Table S2** Primers used for mutant strains verification

| Name | Sequence 5’→3’ |
| --- | --- |
| *glk*-P1 | TCGCCGATTATGCCGAGGTA |
| *glk*-P4 | ACGCTGCTGCCCTCGATGAA |
| *fruA*-P1 | TGTTCCGACGCCGAAGCCGT |
| *fruA*-P4 | CCGCTCCACTCGTGTCGTTC |
| *fruB*-P1 | GCAACATCGGTCCCGCACTC |
| *fruB*-P4 | GGCGCATCTTCCAGCACCTC |
| Over-expression P1 | ACAATTTCCATTCGCCATTC |
| Over-expression P2 | CGTAAATCACTGCATAATTC |
