# Supplementary material for: Improving CoQ10 productivity by strengthening glucose transmembrane of Rhodobacter sphaeroides
Source: Microb Cell Fact. 2021 Oct 30;20:207. doi: 10.1186/s12934-021-01695-z (PMC8557541; doi:10.1186/s12934-021-01695-z)
Supplement: Supplementary file 8 — Additional file 8: Table S3 Primers used for RT-qPCR amplification. [file 12934_2021_1695_MOESM8_ESM.docx]

**Table S3** Primers used for RT-qPCR amplification

| Primers | Sequences (5’-3’) |
| --- | --- |
| 16S-F | GTGSTGCAYGGYTGTCGTCA |
| 16S-R | ACGTCRTCCMCACCTTCCTC |
| *glk*-F | ACGGGGTGGCCCTTCT |
| *glk*-R | CGCCCACGACGAGCAT |
| *fruA*-F | CGGCGTCTACAAGCAC |
| *fruA*-R | TGGAACAGCGTATGGC |
